# Non-Contact Current Measurement for Three-Phase Rectangular Busbars Using TMR Sensors

**DOI:** 10.3390/s24020388

**Published:** 2024-01-09

**Authors:** Huafeng Su, Haojun Li, Weihao Liang, Chaolan Shen, Zheng Xu

**Affiliations:** 1 Dongguan Power Supply Bureau of Guangdong Power Grid Co., Ltd., Dongguan 523120, China; haojunli401@163.com (H.L.); liangwh523@163.com (W.L.); 2 School of Electrical Engineering, Chongqing University, Chongqing 400044, China; 202111131074t@cqu.edu.cn

**Keywords:** non-contact current measurement, TMR sensors, online calibration, rectangular busbars, distribution cabinet

## Abstract

This paper proposes a non-contact current measurement method for three-phase rectangular busbars based on TMR (tunneling magneto-resistance) sensors, due to their advantages of large dynamic range, wide bandwidth, light weight, and easy installation. A non-contact current sensor composed of only three TMR sensors is developed and the TMR sensors are respectively placed at a location with a certain distance from the surface of each rectangular busbar to measure the magnetic fields generated by the busbar currents. To calibrate the developed current sensor, i.e., to establish the relationship between the magnetic fields measured by the TMR sensors and the currents flowing in the three-phase rectangular busbars, we designed a thyristor-controlled resistive load as a calibrator, which is connected to a downstream branch of the distribution cabinet. By switching the resistive load, a calibration current, which can be identified from the background current, is generated in one rectangular busbar and its value is measured at the location of the calibrator, and transmitted wirelessly to the location of the TMR sensors. A new and robust method is proposed to extract the voltage components, corresponding to the calibration current, from the voltage waveforms of the TMR sensors. By calculating the proportional coefficients between the calibration currents and the extracted voltage components, online calibration of the current sensor is achieved. We designed and implemented a current measurement system consisting of a current sensor using TMR sensors, a thyristor-controlled resistive load for current sensor calibration, and a data acquisition circuit based on a multi-channel analog-to-digital converter (ADC). Current measurement experiments were performed in a practical distribution cabinet installed in our laboratory. Compared to the measurement results using a commercial current probe with an accuracy of 1%, the relative error of the measured currents in RMS is less than 2.5% and the phase error is less than 1°, while the nonlinearity error of the current sensor is better than 0.8%.

## 1. Introduction

Distribution cabinet is a key module of the power distribution network to realize power distribution, the current, flowing through the rectangular busbars inside the distribution cabinet, is important information that the power company needs to record. The most commonly used method for measuring busbar current is to use a current transformer based on the principle of electromagnetic induction. However, traditional current transformers have many shortcomings, including their large size, magnetic saturation, and narrow frequency band, which are not suitable for measurement scenarios in compact distribution cabinets. Due to the relatively narrow frequency band of the current transformers, they are not accurate enough for measurement of higher harmonic currents. The phenomenon of magnetic core saturation is prone to occur, leading to an increase in non-linearity [1,2]. Some researchers have also proposed the photoelectric current transformer to overcome the problems of the current transformer based on the principle of electromagnetic induction; however, photoelectric current transformer is not yet widely used due to its high manufacturing and maintenance costs, and susceptibility to the environmental temperature [3].

In recent years, the tunneling magnetoresistance (TMR) sensor has been extensively researched as an innovative current measurement technique due to its significant advantages such as high sensitivity, wide bandwidth, and good linearity [4,5,6,7,8,9,10,11]. Wang et al. proposed a current sensor based on TMR technology, which consists of two TMR sensors to form a differential structure, while offline calibration is required to obtain the proportional coefficients between the measured magnetic fields and the currents [12]. Ma et al. used multiple TMR sensors to form a circular array for measuring the current of a single conductor [13]. Xu et al. used two TMR sensors to form a differential structure and fit them on the rectangular busbar to achieve current measurement over a wide temperature range [14]. Furthermore, Xu et al. also used four TMR sensors placed at different locations near a rectangular busbar, and then realized the measurement of the rectangular busbar current [15]. In all the aforementioned methods, offline calibration is required to determine the linear relationship between the current in the conductor and the measured magnetic fields. Zhang et al. used four three-dimensional TMR sensors to measure the magnetic fields around a single conductor with a circular cross-section, and achieved the accurate position and tilt angle of the measured conductor, and realized the on-line calibration of the current sensor [16]. Liu et al. used seven TMR sensors to form a half-ring structure for measuring the current of a single-phase inlet line, and realized the online calibration of the current sensor [17]. However, the abovementioned online calibration method needs more magnetic sensors to construct a redundant set of equations and realizes inverse computation, significantly increasing the complexity and cost of the whole current sensor system. 

The purpose of this paper is to achieve non-contact current measurement of the three-phase rectangular busbars in a distribution cabinet characterized with a compact space. The mathematic model of the magnetic field inverse problem is more complex, and it is difficult to realize the placement of a large number of TMR sensors in such a compact space. Fortunately, after a thorough investigation of the literature, we found a potential method for our current measurement requirements. Lin et al. employed three magnetic sensors to measure the magnetic fields surrounding a single-phase, three-wire entry line for North American residential customers [18]. Three resistive loads connected to the switches were used to generate an identifiable current in the entry line current such that the online calibration of the current sensor could be achieved. This method has the advantages of requiring less magnetic sensors and low computation power. Considering the above superior performance, in this paper, we explored, for the first time, the application of the above method to the non-contact current measurement for three-phase rectangular busbars in distribution cabinet situations.

In order to achieve high-accuracy current measurement dedicated to the three-phase rectangular busbars, a non-contact current sensor composed of only three TMR sensors was developed and the TMR sensors placed at a certain distance from the surface of each busbar. An online calibration method was used to determine the coefficient matrix corelating the measured currents and the sensed magnetic fields by the TMR sensors. A calibration current, which can be identified from the background current while its value is measured and transmitted wirelessly to the location of the TMR sensors, was alternatively generated in each rectangular busbar using a thyristor-controlled resistive load. The most import point of the calibration method is to accurately extract the voltage components, corresponding to the calibration current, from the voltage waveforms of the TMR sensors, such that the coefficient matrix can be obtained. A novel and robust method was proposed for the above voltage components extraction. Compared to the traditionally used method in [18], no edge detection is required and it has a higher robustness without caring the signal edges submerged by the heavy background current.

The organization of the paper is as follow: Section 1 offers a brief introduction of the development of the non-contact current measurement technique and the purpose, novelty, and contribution of this paper are also presented; An illustration of the non-contact current measurement method for three-phase rectangular busbars is displayed in Section 2; Section 3 presents the optimization of the placement position of the TMR sensors such that a higher noise-to-signal ratio can be obtained; Section 4 shows the details of the circuit design used to construct a non-contact current measurement system; results and discussions are given in Section 5 and finally, the conclusion is drawn in Section 6. 

## 2. Method

### 2.1. The Relationship between the Three-Phase Currents and the Measured Magnetic Fields

**Figure 1:**
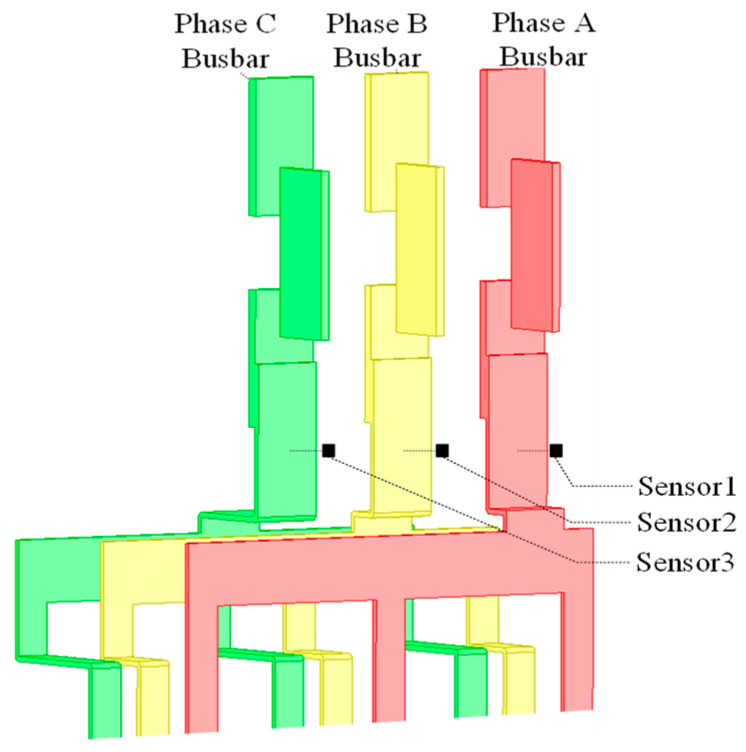
Three TMR sensors (Sensor 1, Sensor 2, and Sensor 3) in front of the rectangular busbars.

Figure 1 shows the structure of the three-phase rectangular busbars in a typical distribution cabinet. Three TMR sensors (Sensor 1, Sensor 2, and Sensor 3) are, respectively, placed in front of each rectangular busbar.

Based on the Biot–Savart law and the superposition law, for one certain harmonic frequency *f*, the current phasors of the three-phase rectangular busbars and the voltage phasors of the TMR sensors satisfy the following relationship:

(1) $$\left[\begin{array}{l} {\dot{S}}_{1,f}\\ {\dot{S}}_{2,f}\\ {\dot{S}}_{3,f} \end{array}\right]=\left(\begin{array}{ccc} k_{1a,f} & k_{1b,f} & k_{1c,f}\\ k_{2a,f} & k_{2b,f} & k_{2c,f}\\ k_{3a,f} & k_{3b,f} & k_{3c,f} \end{array}\right)\left[\begin{array}{l} {\dot{I}}_{a,f}\\ {\dot{I}}_{b,f}\\ {\dot{I}}_{c,f} \end{array}\right]$$

where $[{\dot{I}}_{f}]={\left[{\dot{I}}_{a,f},{\dot{I}}_{b,f},{\dot{I}}_{c,f}\right]}^{T}$ represents a column vector composed of the current phasors flowing in the three-phase rectangular busbars at harmonic frequency *f*, which are the variables to be solved; $[{\dot{S}}_{f}]={\left[{\dot{S}}_{1,f},{\dot{S}}_{2,f},{\dot{S}}_{3,f}\right]}^{T}$ represents a column vector composed of the voltage phasors of the three TMR sensors at harmonic frequency *f*, which are linearly proportional to the measured magnetic fields. The coefficient matrix [*K_f_*], corelating $\left[{\dot{S}}_{f}\right]$ and $\left[{\dot{I}}_{f}\right]$, is a 3 × 3 matrix, whose element may be a frequency-dependent complex number and is related to the relative positions between the rectangular busbars and the TMR sensors. Once the relative positions between the TMR sensors and the rectangular busbars are fixed, then [*K_f_*] remains unchanged. We can calculate the current phasors at harmonic frequency *f* flowing in the rectangular busbars using the following formula:

(2) $${\left[{\dot{I}}_{f}\right]}_{3\times 1}={\left[K_{f}\right]}_{3\times 3}^{-1}{\left[{\dot{S}}_{f}\right]}_{3\times 1}$$

The key issue in achieving current measurement is to determine the coefficient matrix [*K_f_*] for considered harmonic frequencies and the corresponding current phasors can be obtained using (2). Based on the obtained current phasors, time-domain currents, helpful in various applications, such as power measurement and fault diagnosis, can be calculated based on the inverse fast Fourier transform (IFFT) as follows:

(3) $$i_{j}(t)=\sum_{f}\left|{\dot{I}}_{j,f}\right|\cos(2\pi ft+∠{\dot{I}}_{j,f})$$

where *j* represents the phase of the rectangular busbar, i.e., a, b, and c, and $∠{\dot{I}}_{j,f}$ represents the phase angle of the current phasor at harmonic frequency *f*.

### 2.2. Determination of the Coefficient Matrix [K_f_]

**Figure 2:**
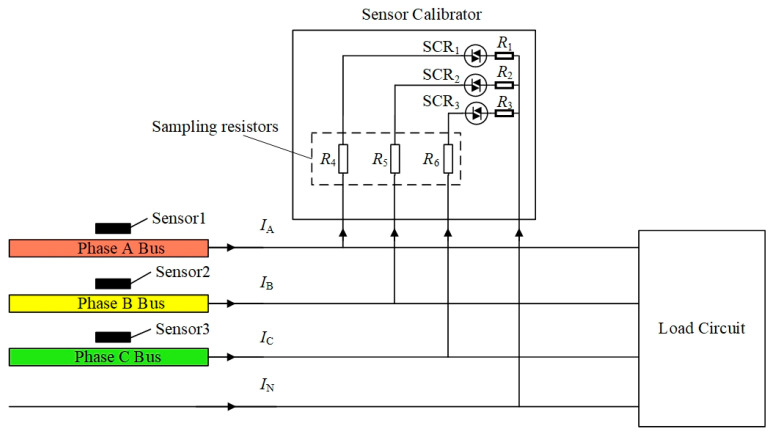
Schematic diagram for calibrating the field-current matrix *K*.

This article uses the method proposed in [18], which is briefly introduced here. As shown in Figure 2, a sensor calibrator, consisting of three resistive loads (*R*_1_, *R*_2_, and *R*_3_) and three thyristors (SCR_1_, SCR_2_, and SCR_3_), and three sampling resistors (*R*_4_, *R*_5_, and *R*_6_), is connected to a downstream branch of the three-phase rectangular busbars in a typical distribution cabinet. The resistive loads are controlled by the thyristors such that a calibration current can be generated in the rectangular busbars.

Taking the rectangular busbar of phase A as an example, when *R*_1_ is connected between the phase A and the N line (*R*_2_ and *R*_3_ are disconnected), the phase A current increases from the original load current *i_a,_*_Load_(*t*) to *i_a,_*_Load_(*t*) + *i_a,_*_cal_(*t*), where *i_a,_*_cal_(*t*) is the calibration current flowing through *R*_1_, while the currents flowing through the two other rectangular busbars remain unchanged. By switching on the thyristor for one cycle and switching off for the next cycle, the calibration current waveform of one power cycle will appear in the total current waveform with an interval of one power cycle, indicating that the calibration current is modulated to the background current, i.e., *i*_a,Load_(*t*). During calibration, the modulation method is alternatively performed for each phase and the corresponding current waveforms *i_j_*(*t*), *j* = a,b,c can be described as below:

(4) $$i_{j}(t)=\left\{\begin{array}{l} i_{j,cal}(t)+i_{j,Load}(t),\;\;\;\;\;\;nT\leq t<(n+1)T\\ i_{j,Load}(t),\;\;\;\;\;\;\;\;(n+1)T\leq t<(n+2)T\;\; \end{array}\right.\;$$

where *n* belongs to an integer number and T represents the power frequency period.

The principle of calibrating the current sensor is described as follows. Taking phase A as an example, the thyristor SCR_1_ is periodically switched on and off with a power frequency duration and the other thyristors, i.e., SCR_2_ and SCR_3_ stay in the off state. Then a calibration current *i_a,_*_cal_(*t*) is generated in the total current waveform in phase A, a voltage waveform with similar shape to the calibration current will be generated in all the TMR sensors. The calibration current is measured using the sampling resistors, as shown in Figure 1, and the corresponding current phasors ${\dot{I}}_{a,cal,f}$ are obtained and transmitted wirelessly to the location of the TMR sensors. The voltage components, induced by the calibration current, are extracted from the voltage waveforms of the three TMR sensors, based on which the corresponding voltage phasors, denoted by $\Delta {\dot{S}}_{1a,f}$, $\Delta {\dot{S}}_{2a,f}$, and $\Delta {\dot{S}}_{3a,f}$, are also obtained. The above procedure is performed similarly on phase B and phase C, respectively. The obtained current phasors and their corresponding voltage phasors of the TMR sensors are, respectively, written in a form of a matrix and represented by (5) and (6):

(5) $${\left[{\dot{I}}_{cal,f}\right]}_{3\times 3}=\left[\begin{array}{ccc} {\dot{I}}_{a,cal,f} & 0 & 0\\ 0 & {\dot{I}}_{b,cal,f} & 0\\ 0 & 0 & {\dot{I}}_{c,cal,f} \end{array}\right]$$

(6) $${\left[\Delta {\dot{S}}_{f}\right]}_{3\times 3}=\left[\begin{array}{ccc} \Delta {\dot{S}}_{1a,f} & \Delta {\dot{S}}_{1b,f} & \Delta {\dot{S}}_{1c,f}\\ \Delta {\dot{S}}_{2a,f} & \Delta {\dot{S}}_{2b,f} & \Delta {\dot{S}}_{2c,f}\\ \Delta {\dot{S}}_{3a,f} & \Delta {\dot{S}}_{3b,f} & \Delta {\dot{S}}_{3c,f} \end{array}\right]$$

Finally, based on (1), (5), and (6), the coefficient matrix [*K_f_*] can be calculated by the following:

(7) $${\left[K_{f}\right]}_{3\times 3}={\left[\Delta S_{f}\right]}_{3\times 3}\times {\left[I_{cal,f}\right]}_{3\times 3}^{-1}$$

Once the calibration is completed, the calibrator can be removed and be used again if recalibration of the current sensor composed of TMR sensors is required. The key for accurate calibration of the current sensor is how to guarantee synchronization between the current phasor of the calibration current and its corresponding voltage phasors of the TMR sensors. This is illustrated in detail in Section 5.1, where a novel and robust method for extracting the voltage components, induced by the calibration current, from the voltage waveforms of the TMR sensors is proposed.

## 3. Sensor Position Optimization

**Figure 3:**
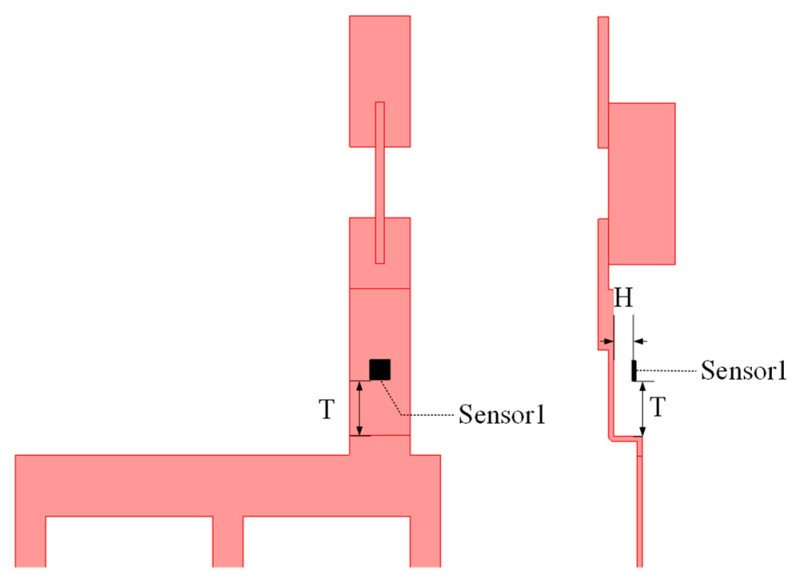
Position of the TMR sensor.

The position of a magnetic sensor is related to the strength of its output signal, so it is necessary to consider the optimal placement of the TMR sensors. Taking the rectangular busbar of phase A as an example, two factors are considered, i.e., the distance H from the TMR sensor to the surface of the rectangular busbar and the distance T between the TMR sensor and the lower end of the rectangular busbar, as shown in Figure 3.

**Figure 4:**
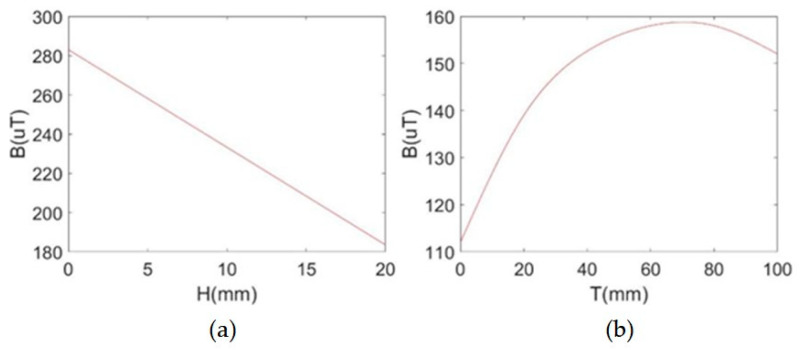
Relationship between the strength of magnetic field and the location of the magnetic sensor. (**a**) Magnetic field versus H. (**b**) Magnetic field versus T.

In order to find the optimal placement location of the TMR sensors, this paper uses the finite element software ANSYS Electronics Desktop 2021 to simulate the magnetic field distribution near the rectangular busbars and the simulated results are shown in Figure 4. As can be seen from Figure 4a, the magnetic field gradually decreases as the distance H between the TMR sensor and the surface of the rectangular busbar increases. The closer it is to the rectangular busbar of the magnetic sensor, the stronger is the measured magnetic field. According to the safety distance regulations of IEC [19], H is selected as 1 mm. From Figure 4b, it can be seen that as the distance T from the TMR sensor to the lower end of the rectangular busbar increases with the magnetic field value and peaks at 70 mm, which is the height chosen for placing the TMR sensor, such that a higher signal-to-noise ratio of the TMR sensors can be obtained.

## 4. Circuit Design

### 4.1. Current Measurement Circuit

**Figure 5:**
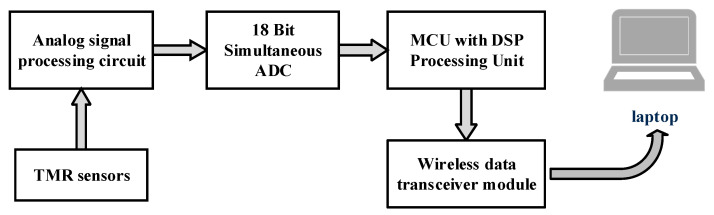
The block diagram of the current measurement system.

The block diagram of the current measurement system is shown in Figure 5. Three TMR sensors are used and each of them is installed at a certain distance from the surface of the corresponding rectangular busbar, and the output voltage signal of each TMR sensor is filtered and amplified by the designed signal processing circuit. Subsequently, the processed voltage signal is sampled by an 18-bit simultaneous multi-channel ADC, the acquired digital signal is processed by the MCU (micro controller unit), and the measured current data is sent to the host computer through the wireless data transceiver module. A detailed description of the key module circuit is displayed as follows.

**Figure 6:**
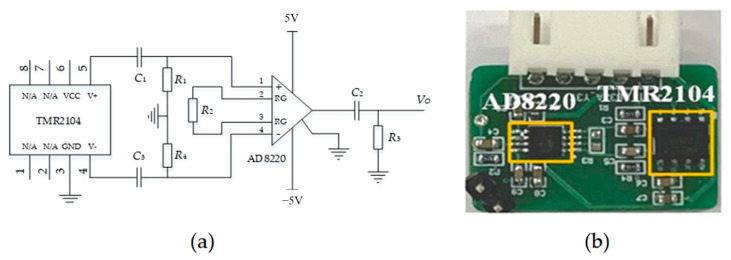
(**a**) Schematic diagram of the TMR sensor and its analog signal processing circuit. (**b**) The designed PCB circuit.

The schematic diagram of the TMR sensor with its analog signal processing circuit is shown in Figure 6, and these two parts are integrated into one single printed circuit board (PCB). The TMR sensor is a TRM2104 from China Jiangsu MultiDimension Technology Co., Ltd. (Suzhou, China) [20]. 

TMR2104 converts the sensed magnetic field into a differential voltage output. In practical work, there is a deviation in the DC components of the two outputs (V+ and V−) of the TMR2104. If it is directly output to the subsequent instrument amplifier, it may result in a large DC component output, thereby reducing the effective dynamic range of the instrument amplifier. Considering that the measured magnetic field is an alternating magnetic field, a high-pass filter can be directly used to filter out the DC component in the output of the TMR sensor. In Figure 6a, resistor *R*_1_ and capacitor *C*_1_, along with resistor *R*_4_ and capacitor *C*_3_, form two high-pass filters that are used to filter out the DC component of the output voltage of the TMR2104. The instrumentation amplifier is AD8220 from ADI, and resistor *R*_2_ controls the gain of the amplifier. AD8220 converts the differential signal into a single-ended signal, which is amplified and filtered using a high-pass filter composed of *C*_2_ and *R*_3_. Figure 6b illustrates the real circuit, characterized with a size of only 15 mm × 22 mm.

**Figure 7:**
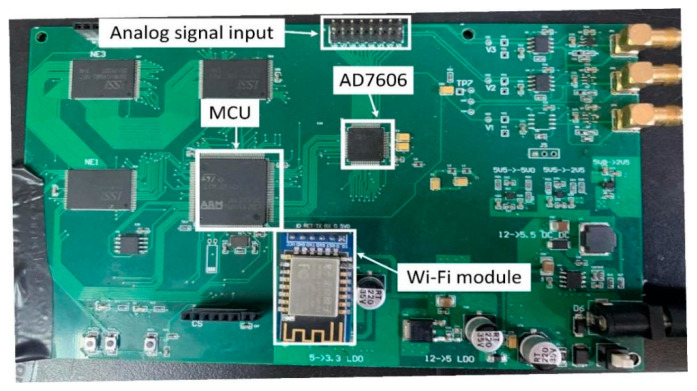
The designed signal conditioning circuit including modules of ADC, microcontroller, and Wi-Fi.

The analog-to-digital converter, the microcontroller, and the Wi-Fi module are integrated into a single PCB, as shown in Figure 7. The ADC chip is an AD7608, which is an 18-bit, 8-channel synchronous sampling ADC. The microcontroller is the ST STM32F407 microcontroller. The wireless data transceiver module supports the standard IEEE802.11b/g/n protocols and the complete TCP/IP protocol stack.

### 4.2. Calibrator Circuit

**Figure 8:**
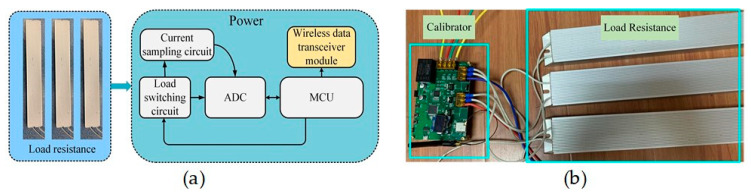
(**a**) Block diagram of the calibrator for calibrating the coefficient matrix *K.* (**b**) Physical drawing of the practically implemented calibrator.

The calibrator circuit is installed in combination with the calibrator resistors as shown in Figure 2. The calibrator circuit is used to switch on/off the three resistors, i.e., *R*_1_, *R*_2_, and *R*_3_, to generate an identifiable calibration current. The block diagram of the associated hardware is shown in Figure 8a and the implemented circuit is shown in Figure 8b. It includes the following parts: the microcontroller circuit, wireless data transceiver circuit, analog-to-digital converter, load switching circuit, current sampling circuit, and calibration resistors. The microcontroller control circuit, ADC, and wireless data transceiver circuit are the same as those used in the current measurement circuit shown in Figure 7. 

The connection and disconnection of the calibration resistors are achieved by using three thyristors controlled by the microcontroller. The thyristors are ACST-610 from STMicroelectronics (ST), which requires only a 10 mA gate current to conduct and can be directly driven by the microcontroller.

The sampling circuit consists of three high-precision current sampling resistors and three amplifiers, which are used to measure the calibration currents. The sampling resistor, i.e., *R*_4_, *R*_5_, and *R*_6_ in Figure 2, is a high-precision resistor with a resistance value of 15 milliohms and an accuracy of 0.1%.

## 5. Results and Discussions

**Figure 9:**
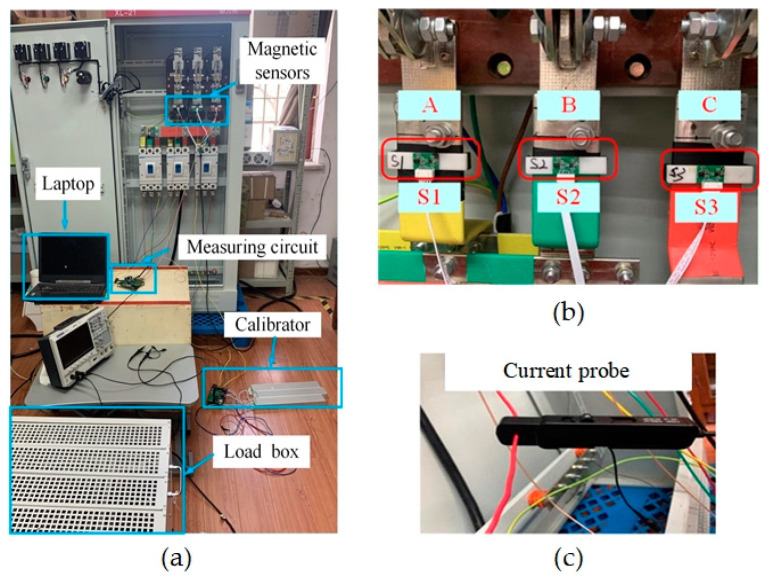
(**a**) The constructed experimental platform. (**b**) The installed TMR sensors. (**c**) The commercial current probe for reference current measurement.

A measurement platform is constructed in our lab and current measurement experiments are performed to verify the validity of the proposed non-contact current measurement method for three-phase rectangular busbars. Figure 9a shows the overall view of the experimental platform. The three-phase rectangular busbars in the distribution cabinet are connected to the three-phase power lines, and three TMR sensors are installed on the three-phase rectangular busbars of phase A, B, and C, respectively, and a three-phase simulation load is connected to the three-phase rectangular busbars of the distribution cabinet to simulate the practically operating load or the background load. The calibrator is also connected in the system, in parallel with the simulated load. The calibrator is connected to the circuit, only when calibration of the current sensor is required, and is removed when the calibration is completed. Figure 9b shows the magnetic sensor installation in situ with three TMR sensors (S1, S2, and S3) mounted on the surface of the three-phase rectangular busbars. The currents flowing through the three-phase rectangular busbars are also measured synchronously as a reference with three Pintech current probes with a product NO. of PT-320 having an accuracy of 1%.

### 5.1. Sensor Calibration

**Figure 10:**
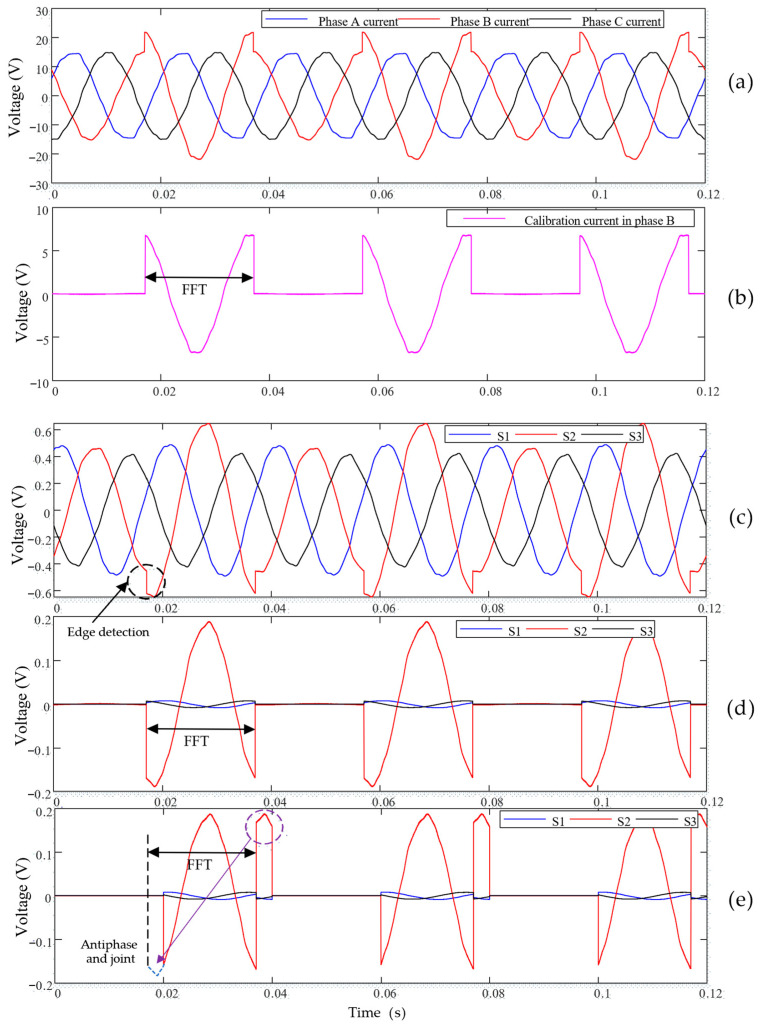
(**a**) Three-phase currents flowing through the three-phase rectangular busbars during calibration. (**b**) Calibrating current imposed in the rectangular busbar of phase B. (**c**) Voltage waveforms of the TMR sensors. (**d**) Extracted voltages of the TMR sensors induced by the calibration current using the method in [18]. (**e**) Extracted voltages of the TMR sensors induced by the calibration current using the newly proposed method in this paper.

The key point to realize calibration of the developed current sensor is to generate an identifiable calibration current in the rectangular busbar of each phase alternatively by using a calibrator controlled by thyristors. The current phasor of the calibration current and the corresponding voltage phasors of the TMR sensors should be obtained synchronously. Then the coefficient matrix can be calculated using (7). The principle of calibrating the current sensor is comprehensively illustrated using Figure 10. The calibrator is connected to phase B and the three-phase currents flowing in the three-phase rectangular busbars are acquired, as shown in Figure 10a. The corresponding calibration current flowing through the rectangular busbar of phase B is also obtained, as shown in Figure 10b, and the thyristor is switched at approximately the peak point of the calibration current. FFT analysis is performed on one complete cycle of the calibration current, as shown in Figure 10b, and the current phasor ${\dot{I}}_{b,cal,f}$ is obtained. 

Figure 10c shows the acquired voltage waveforms of the three TMR sensors and for TMR sensor S_2_, an edge is clearly visible caused by the switching of the thyristor of the calibrator, However, the signal edges are not sufficiently obvious for the TMR sensors S_1_ and S_3_, because the magnetic fields generated by the calibration current at the location of S_1_ or S_3_ are very weak. In order to extract the voltage component, induced by the calibration current, from the voltage waveforms of the TMR sensors, the commonly used method [18] is to detect the edge point, as shown in Figure 10c. The voltage component is then extracted by subtracting one power cycle of the voltage waveform of one specific TMR sensor with a closely followed cycle, and each of the two adjacent cycles is limited between two detected edges. The results of the extracted voltage components are shown in Figure 10d for the three TMR sensors and FFT analysis is performed on one certain complete cycle of the extracted waveforms to obtain the voltage phasors $\Delta {\dot{S}}_{1b,f}$, $\Delta {\dot{S}}_{2b,f}$, and $\Delta {\dot{S}}_{3b,f}$. As can be seen, the commonly used method [18] requires accurately detecting the signal edges, as shown in Figure 10c; however, usually the calibration current is much smaller than the background current and the signal edges may be submerged, causing a decrease of the accuracy of the extracted voltage components. 

In order to solve the above problem, in this paper, a new and more robust method is proposed. Instead of detecting the signal edges first, we directly use one complete cycle of the voltage waveforms, as shown in Figure 10c, to subtract the previous cycle. The subtracted results are shown in Figure 10e and, as can be seen, one complete cycle of the extracted results consists of two parts, and by inverting the second part with an angle of 180° and re-connecting it to the first part, indicated by the purple arrow, a new cycle can be obtained, which is the same as the results shown in Figure 10d. Finally, FFT analysis is performed on the newly acquired one complete cycle of the voltage components and the corresponding voltage phasors can be obtained.

The traditional method for extracting the voltage component, caused by the calibration current from the voltage waveforms of the TMR sensors, is highly dependent on accurately detecting the signal edges of the voltage waveforms of the TMR sensors. Thus, there should exist obvious edges or steps in the calibration current, as shown in Figure 10b, which is achieved by switching the calibration load at the time instant of the peak value of the phase voltage waveform measured by a voltage sensor. Thus, additional voltage sensors and real-time detection of the time instants of the phase voltage waveform significantly increase the cost and complexity of the current measurement system. It is obvious that the traditional method will lose its efficacy under the condition that the calibration load is periodically switched at the zero-crossing instant of the phase voltage waveform, resulting in no edges existing in the calibration current. However, the newly proposed method can still successfully extract the voltage component associated with the calibration current from the voltage waveforms of the TMR sensors without detecting the edges, indicating that the calibration load can be periodically switched at a random time instant. Then, voltage waveform measurement and its peak value detection are not required, which significantly reduce the cost of the calibration device and the complexity of the calibrating procedure. So, the newly proposed method is much more robust than the traditional method. 

### 5.2. Current Measurement

**Figure 11:**
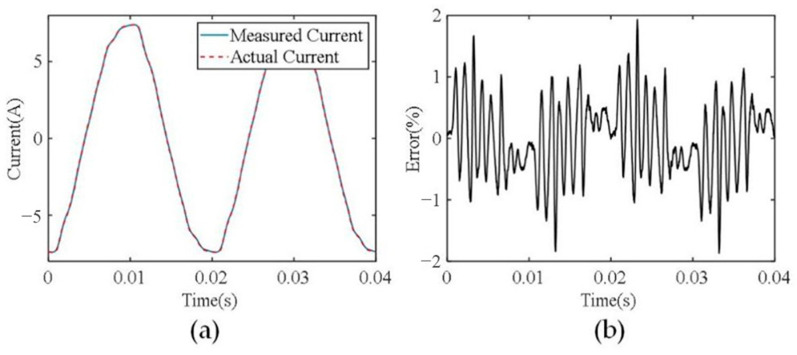
(**a**) The measured current waveforms with an amplitude of 7 A. (**b**) The instantaneous relative errors.

**Figure 12:**
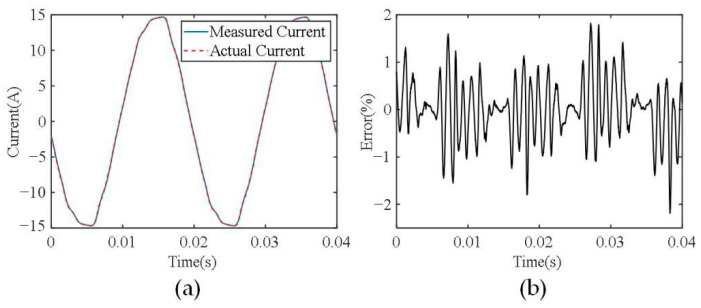
(**a**) The measured current waveforms with an amplitude of 15 A. (**b**) The instantaneous relative errors.

After the current sensor is calibrated, current measurement experiments are carried out to evaluate its current measurement accuracy. Different current levels are provided using a variable simulation load and the generated currents are also measured using commercial current probes of the Pintch PT-320 as reference. Figure 11 and Figure 12 show the measurement results of phase A current, and the currents of the other two phases are similar. The current waveforms shown in Figure 11a have an amplitude of 7 A, where the blue solid line is the measured current waveforms using the developed current sensor and the red dashed line denotes the measured current waveforms using the commercial current probe PT-320. It can be seen that the two waveforms are almost consistent. The instantaneous relative error curve of the two waveforms is shown in Figure 11b, which demonstrates that the maximum instantaneous relative error is less than 2%. Figure 12 shows the measurement results with a current amplitude of 15 A. The maximum instantaneous relative error of the two current waveforms is less than 2.3%. 

**Figure 13:**
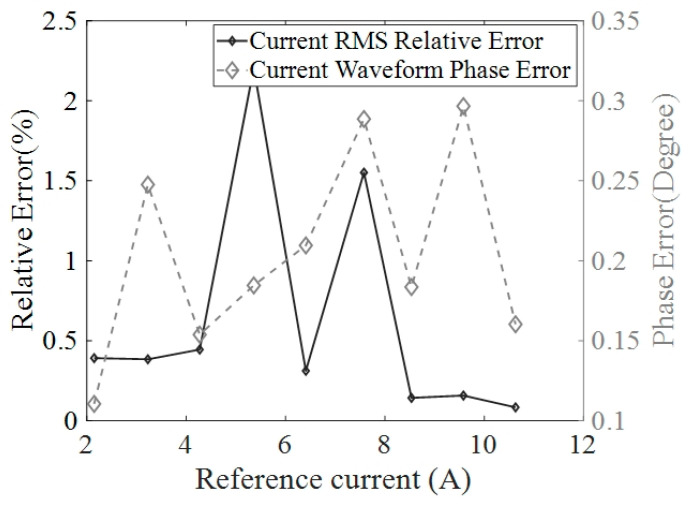
The tested relative errors of the current in RMS and the phase errors referring to the current measurement results using a commercial current probe with an accuracy of 1%.

To further test the measurement accuracy of the developed current sensor, currents in the range of 2 A to 10 A in RMS with a step of 1 A are generated by varying the resistance value of a load connected to the three-phase rectangular busbars. Three commercial current probes PT-320 are used to simultaneously measure the testing currents for comparison. The testing results of the relative errors of the current in RMS and the phase errors for phase A are depicted in Figure 13 and the results for the two other phases are similar. As can be seen from Figure 13, the relative errors of the current in RMS are lower than 2.5% compared to the reference currents measured by the commercial current probe PT-320 while the phase errors are no more than 1°, significantly demonstrating the satisfactory measurement accuracy of the developed current sensor. 

### 5.3. Non-Linearity Evaluation of the Developed Current Sensor

**Figure 14:**
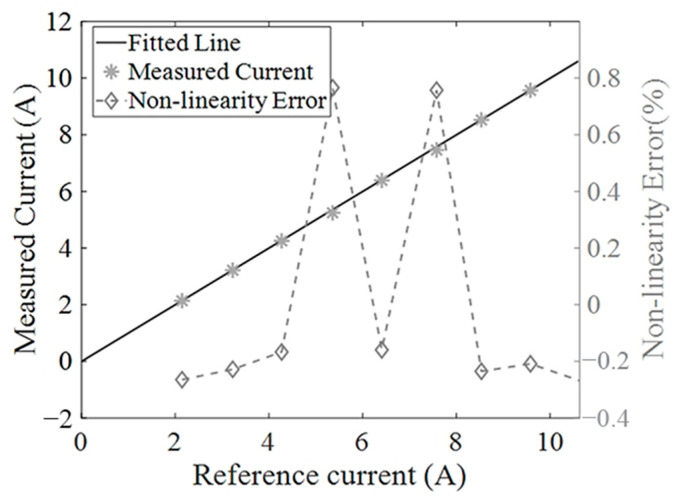
The tested non-linearity errors of the developed current sensor.

Non-linearity is a key parameter to evaluate the performance of the developed current sensor. In order to test the non-linearity of the current sensor, currents approximately varying from 2 A to 10 A in RMS with a step of 1 A are generated in the three-phase rectangular busbars and the current probes PT-320 are also used to measure these currents as a reference. The measurement results by the developed current sensor are given in Figure 14 denoted by the asterisks. The solid line represents the fitted curve based on the current measurement results, demonstrating a high linearity. The relative errors between the practically measured currents and the fitted value are also given in Figure 14, denoted by the diamonds, verifying that the non-linearity error of the designed current measurement system is less than 0.8%. 

### 5.4. Harmonic Interference and Noise Immunity Test

**Figure 15:**
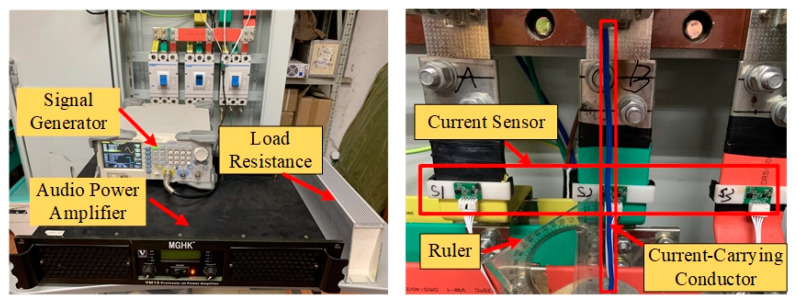
Experimental platform for performing the harmonic interference and noise immunity test.

Harmonic interference experiments were conducted in the laboratory. Figure 15 shows the experimental platform. The RIGOL DG1032 signal generator outputs a sine signal with a frequency of 150 Hz and transmits it to an audio power amplifier. The power amplifier amplifies the signal and drives a resistive load to generate a harmonic current at 150 Hz in a movable conductor, which is parallel to the rectangular busbars such that a maximum harmonic interference is imposed on the current sensor. The current carrying conductor is placed approximately 3–12 cm away from the rectangular busbar of phase B to observe the impact of the harmonic magnetic fields on the current measurement results.

When conducting harmonic interference experiments, three-phase currents are applied to the three-phase rectangular busbars. A commercial current probe is used to measure the current flowing in the rectangular busbar of phase B and a harmonic component of 0.335 A at 150 Hz in the current is obtained by performing FFT analysis. Variable degrees of harmonic magnetic field interferences are introduced by adjusting the distance between the current carrying conductor and the rectangular busbar of phase B. After introducing harmonic interference, the measured 150 Hz harmonic component for phase B and the corresponding relative errors are shown in Table 1. It can be seen that the harmonic interferences have a significant impact on the measurement accuracy of the harmonic current component. The main reason is that the harmonics in the measured current are extremely small. Slight harmonic interference can bring large measurement errors, and during the harmonic interference experiments, the impact of harmonics on the measurement results of the fundamental component of the current of phase B can be ignored.

Then, magnetic field interferences at 50 Hz were generated using the same procedure for generating 150 Hz harmonic interferences and noise immunity experiments were subsequently conducted in the laboratory. Three-phase currents with a fundamental current component of 14.75 A were applied to the three-phase rectangular busbars. The current carrying conductor with a current of 1 A was moved apart from the phase B busbar in a range of 3–12 cm to investigate the impact of the strength of noise interferences on the current measurement accuracy. The experimental results for the fundamental current component of phase B are shown in Table 2. It can be seen that the noise interference at 50 Hz has a significant impact on the measurement results, and as the distance between the current carrying conductor and the current sensor decreases, this effect becomes more significant, resulting in larger measurement errors (maximum 5.56%). The method proposed in this paper does not have a powerful anti-interference ability against external interference, which is also the point needed to be improved in the future. 

### 5.5. Error Analysis 

The current measurement error can be mainly attributed to two aspects: one is from the calibration error and the other is from the electromagnetic interference. The electromagnetic interference will result in a significantly large measurement error. In the research area of non-contact current measurement using a number of magnetic sensors (called magnetic sensor array), the differential mechanism of using two closely placed magnetic sensors in parallel is commonly used to reject the uniform component of the interference electromagnetic field [21]. However, gradient magnetic fields of higher orders are difficult to be fully rejected and at the same time, more magnetic sensors are required resulting in higher complexity of the current measurement system. The simplest and most effective method to reject the electromagnetic interference is to use a magnetic shielding material with high permeability but low conductivity, which will be explored in future work.

Calibration error is the other main source of current measurement error. The coefficient matrix [*K_f_*] is calibrated using Equation (7), thus its accuracy is highly dependent on the measurement errors of the calibration current phasors $\left[{\dot{I}}_{cal,f}\right]$ and the corresponding extracted voltage phasors $\left[\Delta {\dot{S}}_{f}\right]$ of the magnetic sensors, which are manually introduced using a thyristor-controlled resistive load connected to a downstream branch of the three-phase rectangular busbars, as shown in Figure 2. The phasors, i.e., $\left[\Delta {\dot{S}}_{f}\right]$ and $\left[{\dot{I}}_{cal,f}\right]$, are obtained using the fast Fourier transform (FFT) method and the calculation errors can approach a very low level of 10^−4^~10^−5^. Three commercial sampling resistors, as shown in Figure 2, with an accuracy of 0.1% are used to measure the three-phase reference currents flowing through the three-phase resistive loads. A multi-channel ADC (AD7606) with a voltage amplitude measurement error level of 10^−4^ is used to simultaneously sample the time-domain voltages of both the magnetic sensors and the sampling resistors. The measurement error of the reference current is obviously higher than the other errors from the phasor calculation and the voltage signal sampling. Thus, in order to achieve a higher accuracy of the coefficient matrix [*K_f_*], it is better to use current measurement techniques with higher accuracy for reference current measurement during calibration.

Because the coefficient matrix [*K_f_*] is calibrated at a single point, for example, at a current amplitude of 10 A.T, in order to obtain a higher current measurement accuracy, magnetic sensors with better non-linearity characteristic should be used. In this paper, TMR sensors with a product NO were used. TMR2104 has a stated non-linearity of less than 1.5%. After the coefficient matrix [*K_f_*] is determined, the current measurement experiments for evaluating the non-linearity of the developed current sensor were performed and the experimental results are presented in Figure 14. As can be seen, the non-linearity error is in a range of 0.4%–0.8%, which is at the same level of that of the magnetic sensor used. Thus, magnetic sensors with lower non-linearity should be chosen in designing a current sensor to further improve its current measurement accuracy. 

## 6. Conclusions

This paper proposes a non-contact current measurement method for use in the situation of three-phase rectangular busbars in a distribution cabinet. A non-contact current measurement system was designed, which is composed of a current sensor using only three TMR sensors, a thyristor-controlled calibration load used to generate calibration current capable of being identified from the background current, and a data acquisition circuit based on a multi-channel ADC. The procedure of calibrating the current sensor is to determine the coefficient matrix correlating the measured currents and the magnetic fields sensed by the TMR sensors. In order to realize the calibration of the current sensor, a calibration current is alternatively generated in the rectangular busbar of each phase by connecting the thyristor-controlled resistive load to a downstream branch of the distribution cabinet; the calibration current is measured at the location of the calibration load and transmitted wirelessly to the location of the TMR sensors. The voltage components, corresponding to the calibration current, are extracted using a newly proposed method, which is more robust compared to the traditionally used method, and may be inaccurate when the signal edges, in the voltage waveforms of the TMR sensors, are submerged by a heavy background current. The experimental study was performed and the results show that, compared to the measurement results using a commercial current probe with an accuracy of 1%, the relative error of the measured currents in RMS is less than 2.5% and the phase error is less than 1°, while the nonlinearity error of the current sensor is better than 0.8%.

This measurement system still has some shortcomings, which will direct us to conduct further research essentially to improve its performance. Limited by the test capability of our laboratory, the current sensor is experimentally demonstrated with currents of small amplitude. In the future, we will use a large-capacity simulation load to verify the accuracy of the current measurement system for measuring large currents. Sampling resistors with higher accuracy and TMR sensors with better linearity will be selected in the future current measurement system design for better current measurement performance. The non-contact current measurement method may be sensitive to interference from the external magnetic fields, thus, we will perform interference rejecting experiments and by analyzing the results, propose new methods to strengthen the anti-interference ability of the current sensor, such as by adding a magnetic shield structure around the TMR sensors.

## Figures and Tables

**Table 1:** Measured values of harmonic components and corresponding relative errors.

Distance (cm)	12	9	6	3
Amplitude of harmonic component at150 Hz (A)	0.256	0.201	0.145	0.478
Relative Error (%)	−20.9	−40.0	−56.7	42.68

**Table 2:** Experimental results for the fundamental wave current component.

Distance (cm)	12	9	6	3
Amplitude of fundamental component (A)	14.83	14.91	15.16	15.57
Relative error (%)	0.54	1.08	2.78	5.56

## Data Availability

The data presented in this study are available on request from the corresponding author.
